# Effect of red koji as a Starter Culture in “Wanergao”: A Traditional Fermented Food in China

**DOI:** 10.1002/fsn3.1849

**Published:** 2020-09-01

**Authors:** Xuefeng Zeng, Zhongyue Tang, Wei Zhang, Lapin He, Li Deng, Chun Ye, Jin Fan

**Affiliations:** ^1^ School of Liquor and Food Engineering Guizhou University Guiyang China; ^2^ College of Food Science and Engineering Wuhan Polytechnic University Wuhan China

**Keywords:** color, koji, monascus, sensory, Wanergao

## Abstract

The objective of this study was to explore the effect of red kojis on essential indices of Wanergao. The results showed that red koji‐inoculated Wanergao showed higher pH values (4.38 ± 0.06 and 4.39 ± 0.06) and lower TA values (1.61 ± 0.05 and 1.63 ± 0.05) compared to the control group. LAB and yeast in the starter culture group gradually increased to 7.57 ± 0.12, 7.64 ± 0.15 log cfu.g^−1^ and 8.59 ± 0.21, 8.64 ± 0.23 log cfu.g^−1^, respectively. During fermentation, the dominant microorganism was Lactobacillus plantarum and Saccharomyces cerevisiae. Compared to the Wanergao made using traditional backslopping, the red koji‐inoculated Wanergao contained more amylases, EAA and DAA contents compared to the control sample. The red kojis and control samples presented different hardness, chewiness, and cohesiveness, as well as similar values in springiness, gumminess, and adhesiveness. Sensory analysis also showed higher chewiness aroma and resilience of Wanergao in the starter culture group than in the control group.

## Graphical abstract

Sensory characteristics Wanergao with/without starters. Opened round, CN: no starter added; closed square, SM1 starter culture; closed rhombus, SM2 starter culture

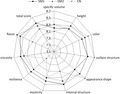

## 1 INTRODUCTION

Color is an important sensory quality index of food, which is also an important factor for attracting consumers. Various types of synthetic and natural colorants all have good color and luster, which can give food sensory beauty and are thus widely used in the food industry (Chen, G., Bei, Q., Huang, T., Wu, Z. J., 2018). However, due to potential safety hazards, the types and quantity of synthetic colorants have been strictly restricted. Research and applications of natural colorants, on the other hand, have gained widespread global attention at present due to their high safety (Shen & Chen, 2013). Thus, development and research of natural colorants have been a very urgent task.

Originating in China, some red koji with monascus and yeast as a natural colorant and starter culture. Microorganisms in red koji mainly include monascus, saccharomycetes and some bacteria, which has advantages unparalleled by many other natural colorants such as pH stability, high capacity strong fermentation and heat resistance. Consumption of red koji is increasing year by year, which has gained widespread use in the wine, red preserved bean curd, meat, and aquatic products at present (Zhao, Jiang, Xu, & Xia, 2017). In the application in fermented foods, the microorganisms and enzymes red koji contains mainly have saccharomycetes, fermenting, and various flavor producing effects, which thereby offer special flavors, healthcare benefits and texture properties to fermented foods (Wang, J., Shen, Y., Huang, Y., Weijie, L. U., & Cheng, B. I., 2013). Besides, red koji can produce a variety of enzymes during growth. When used in the production of starchy foods or meat products, the amylase, glucoamylase, etc. it secretes can hydrolyze starch to form low molecular substances like dextrin, oligosaccharides and glucose to improve the flavor of products. Meanwhile, proteases it secretes can break proteins into polypeptides, amino acids, and other small molecular compounds, so that products can have delicate texture, smooth taste, rich flavor, and reddish color (Atieh, S., Fatemeh, Y., Ashrafalsadat, Behnam, & Mohammad, 2015). In addition, monascus also has healthcare and antibacterial effects. Studies have shown that the monascus has an anti‐Clostridium botulinum activity; besides, monacolin K, GABA, and other metabolites produced also have hypotensive, hypolipidemic, antioxidant, anticancer, and other healthcare functions (Zhao et al., 2017).

Selim Silbir studies the production of natural red pigments by Monascus purpureus CMU001 in the submerged fermentation system using a brewery waste hydrolysate (Silbir & Goksungur, 2019). Beer brewed this way had unique flavor, attractive reddish color, delicate foam, and clear taste. After adding tofu with marinade prepared with monascus and other spices in a certain proportion, colorants and a variety of enzymes in the monascus gave tofu attractive surface color and rich aroma during the later fermentation process (Bodor, Unilever, Vlaardingen, Trautwein, Unilever, & Vlaardingen, 2007). Monascus can replace nitrite in sausage making, and the fermented sausages made this way does not discolor in one month at low temperature storage (Mamucod & Dizon, 2014).

As a traditional fermented rice product widely popular in China's western Hunan and Guizhou, Wanergao is favored by the majority of consumers with its soft taste, unique flavor, and certain healthcare functions (Wu,Xu, Xu, Chen, & Pan, 2011). However, Wanergao is either naturally fermented or artificially fermented by inoculating backslopping. Complex microbial system and easy changes in microbial activity and proportion during fermentation process, as well as susceptibility to bacterial contamination, lead to lengthened production cycle of Wanergao and low level of its industrialization. Technological bottleneck restricting the industrialized production of traditional fermented food Wanergao is the lack of suitable starter culture. Red koji not only contains rich, stable microbial system, but also offers foods vivid color and good healthcare functions, which has thus been widely used in the fermentation of starchy foods, tofu, and fish products (Gum, Nguyen, Lee, Han, & Cho, 2017; Liu et al., 2019; Wei et al., 2013). Therefore, we speculate that red koji as starter culture can well meet the requirements of industrial fermentation of Wanergao, not only opening up new ways to utilization of monascus and industrial production of Wanergao, but also meeting people's needs for healthy eating.

The purpose of this study was to study the effects of red koji as a starter culture in Wanergao, in order to explore the changes in number and types of microorganisms, fermentation performance, color, textural characteristics, nutritional composition, and sensory properties of inoculated Wanergao in the fermentation process and to identify the effects of red koji on improving the quality characteristics of fermented Wanergao.

## 2 MATERIALS AND METHODS

### 2.1 Proliferation of red koji

In the experiment, SM1 and SM2, two red koji (Like uncooked materials koji and like liqueur koji, respectively), were purchased from Lishui like biotechnology Co., Ltd (Lishui city, Zhejiang province), which was used as the starter culture. Accurately weigh 600 g of rice adds an appropriate amount of water and soak it for 24 hr at room temperature and then drain it, steam it, cool it to room temperature, mix it with 15 g of red koji under aseptic conditions, and finally close and ferment it at room temperature for 10–15 days, forming a rich flavor of red koji rice wine that can be used as a starter for producing “Wanergao.” Appearance standards after fermentation: floating starter granules, with small bubbles on the surface (fermentation phenomenon), as well as slight fumes of alcohol.

### 2.2 Preparation of samples

A. Basic ingredients: 100 kg of early indica rice or stale rice, 20 kg of sucrose.

**Figure 1:**
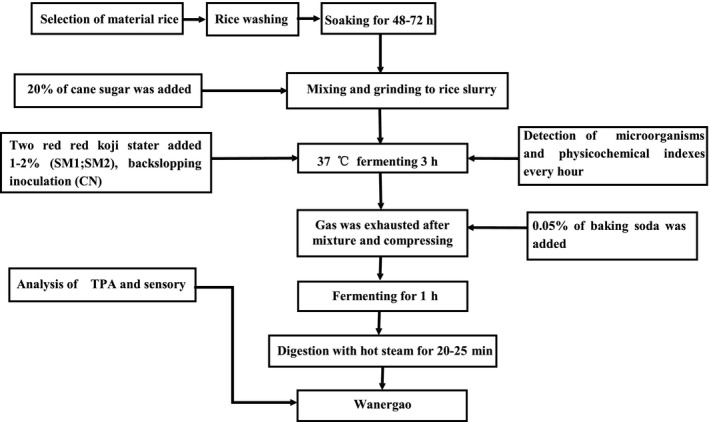
Process flow diagram of Wanergao production

B. Wanergao making process:

Manufacturing flow of “Wanergao” is shown in Figure 1.

In the experiment, samples inoculated with backslopping served as the control group. During the fermentation, samples were collected at a 2‐hr interval for quality analysis.

### 2.3 Microbial detection

20 g of sample was collected aseptically, ground, added into 180 ml of sterile peptone (0.1%) solution, and shaken for 30 min. 1 ml of supernatant was drawn and subjected to 10‐fold gradient dilution. Three replicates of three appropriate dilutions were poured into plates and cultured using selective media. Aerobic bacteria were cultured in nutrient agar at 37℃ for 2 days for the determination of aerobic plate count (APC); lactic acid bacteria (LAB) were cultured in MRS medium at 37℃ for 2 days; enterobacteriaceae were cultured in VRBD agar medium at 37℃ for 2 days; pseudomonadaceae were cultured in GSP agar medium at 26℃ for 3 days; and saccharomycetes and monascus were cultured in YM agar medium at 28℃ for 4 days.

Among dominant strains isolated from Wanergao at different stages, those with highest dilution gradient were selected and purified in the above media. DNA of lactic acid bacteria was extracted by Benito et al. (2008) method, and specific bacterial species were analyzed by 16sRNA gene sequencing. DNA of saccharomycetes was extracted by Kurtzman and Robnett (1998) method, and their bacterial species were analyzed by 26sRNA gene sequencing.

### 2.4 Determination of pH and titratable acidity (TA)

pH: 10 g of Wanergao was taken, homogenized with 90 ml of water, and then measured with a ZD‐2 automatic potentiometric titrator.

TA: 5 g of sample was weighed, homogenized with a small amount of carbon dioxide‐free distilled water, moved into a 250‐ml flask, and heated in a 75–80℃ water bath for 30 min. After cooling, dilution to a constant volume and filtration (discarding initial solution), an appropriate amount of filtrate was taken and titrated with 0.1 M standard NaOH solution, where TA was expressed as lactic acid.

### 2.5 Determination of characteristic rice slurry

Protein content determined according to the method in Latimer (2012). Total starch content determined by acid hydrolysis 3.5‐dinitrosalicylic acid colorimetry according to Latimer (2012). Amylose content determined by rice grain amylose content assay in Latimer (2012).

### 2.6 Increase in batter volume

30 g rice wine was added and mixed into 270 g rice slurry. The mixed rice slurry was then placed in a 500‐ml graduated cylinder, sealed with plastic wrap, and stood still in a 30℃ environment. Expanding volume of mixed rice slurry and amount of CO_2_ generated were recorded hourly.

### 2.7 Bulk density

Changes in bulk density of Wanergao (g/m^3^) were determined by mustard seed displacement method used for analyzing bulk density of bread. Volume change determination formula was as follows:

$$\text{Specific}\;\text{volume}=\frac{\left[\left(\text{Initial}\;\text{volume}\;\text{of}\;\text{mustard}\;\text{seeds}\right)‐\left(\text{Volume}\;\text{of}\;\text{mustard}\;\text{seed}\;\text{after}\;\text{displacement}\;\text{with}\;\text{product}\right)\right]}{\text{Weight}\;\text{of}\;\text{the}\;\text{product}}$$

Bulk density = 1/Specific volume.

### 2.8 Determination of textural properties of Wanergao

Wanergao was placed on slicer, so that the bottom of Wanergao was perpendicular to the slicer edge. After powering on the slicer, Wanergao was cut into 12.5‐mm‐thick slices. Two Wanergao were taken from each batch of samples as parallel samples. Two middle slices were taken from each Wanergao and tested using a texture analyzer with P35 cylindrical indenter. Testing was repeated four times and averaged.

TPA mode was adopted; testing parameters were as follows: compression ratio: 40%; pretest speed: 3 mm/s; test speed 1 mm/s; and post‐test speed 5 mm/s.

### 2.9 Determination of free amino acid content

Appropriate amount of sample was accurately weighed, placed in hydrolysis tube, added with 6 M hydrochloric acid solution, vacuum sealed, and hydrolyzed at 110℃ for 24 hr. After cooling, the hydrolysate was diluted to constant volume, filtered, evaporated to dryness, added with 0.02 M hydrochloric acid solution, and placed in the air for 30 min, followed by the determination of free amino acid content with HPLC system (HP1100, Agilent, USA). Peak identification and quantification were completed by determining the retention time and recoveries of FAA standards (Sigma Chemical, St Louis, MO, USA).

### 2.10 Color value analysis

To make the absorbance within a 0.2–0.7 range, twofold dilution was adopted according to the absorbance spectrum of Monascus pigments at their characteristic wavelength (Bodor et al., 2007). Specifically, 5 g of Wanergao was ground with a mill, added into a small conical flask containing 10 ml of 70% ethanol solution, shaken in a constant temperature water bath oscillator for 2 hr, and then filtered for colorant extract through four layers of gauze. Afterward, the extract was placed in a 1‐cm cuvette and measured for absorbance at 505 nm with a UV spectrophotometer. 70% ethanol solution served as the blank control.

Color value (CV) = OD_505_ × dilution factor (unit: U/g).

Dilution factor was 2 in this experiment.

### 2.11 Qualitative analysis of citrinin

0.1 g of samples at different fermentation periods was accurately weighed, dried to constant weight at 45℃, placed in a stoppered test tube, added with 10 ml of methanol, and then vibration extracted in a 60℃ constant temperature water bath for 1 hr. After centrifugating at 6,000 r/min for 10 min, supernatants were collected as test samples. Samples were dripped on plates with capillary tube; meanwhile, citrinin standard was developed with a developing solvent (toluene: ethyl acetate: formic acid = 6:3:1). After development, the plates were observed under UV lamp with standard as control. Sample spots with Rf value similar to standard were precisely citrinin in samples.

### 2.12 Sensory evaluation

Wanergao were scored for several edible quality indices such as color, shape, taste, flavor, and mouthfeel by fifteen people according to the sensory evaluation criteria. Sensory evaluation methods: Color and shape were evaluated by observation of samples. Samples were placed in a well‐lit place for careful observation of luster, color, and shape. Evaluation of flavor: Samples were placed about 5 cm directly below the nose and inhaled deeply 3–5 times for careful identification of the flavor released. Evaluation of taste: Samples were placed in the mouth, chewed carefully, and swallowed slowly 3–5 times for identification of taste. Evaluation of mouthfeel: Samples were placed in the mouth and chewed carefully, so that they were full of mouth, which was repeated 3–5 times for identification of hardness, viscosity, chewiness, and smoothness. These indices and their score ranges were as follows: surface specific volume, height, color, surface structure, appearance shape, internal structure, elasticity, resilience, viscosity, flavor and a total score. The full mark was 9, that is, 1 represented worst, 5 indicated moderate, and 9 was the best.

## 3 RESULTS AND DISCUSSION

### 3.1 Microbial analysis

**Figure 2:**
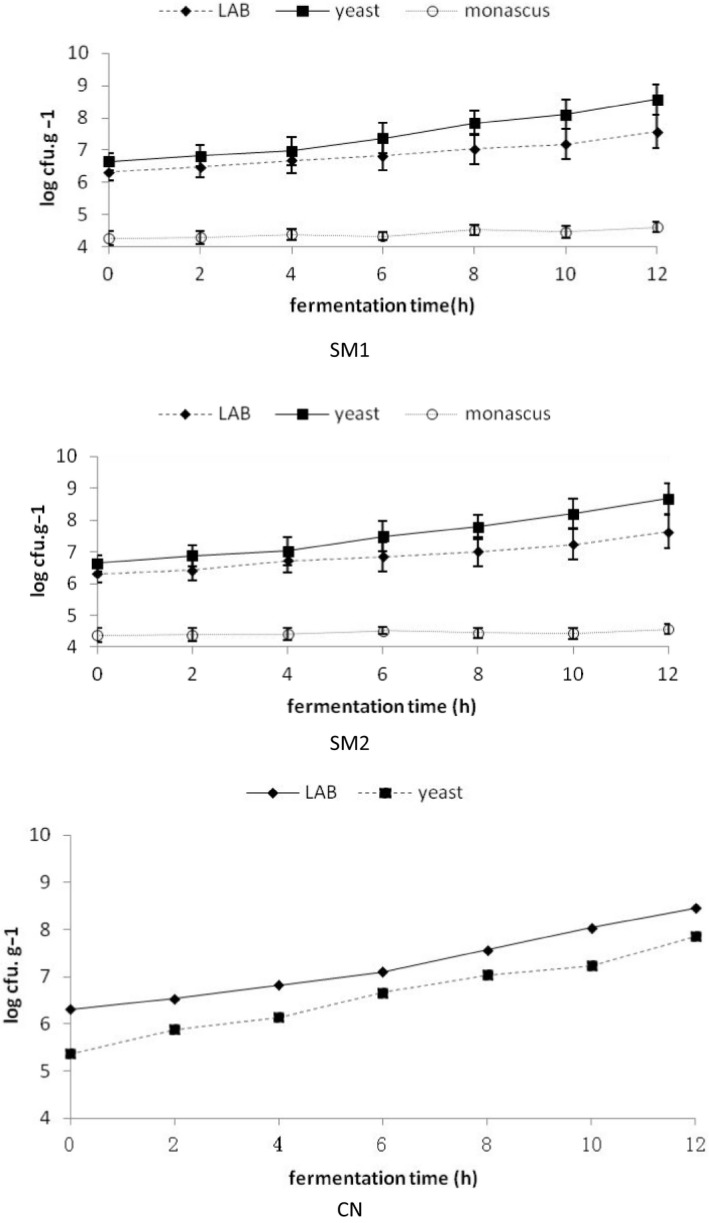
Microbiological changes in Wangergao with/without starters duringfermentation. Opened round, monascus; Closed square, yeast; Closed rhombus, LAB

Changes in the number and types of lactic acid bacteria and saccharomycetes during Wanergao fermentation are shown in Figure 2. As can be seen from the Figure 2, in the starter culture group, lactic acid bacteria and saccharomycetes in the two groups of samples all exhibited rapid growth trend in the fermentation process, while the amount of monascus hardly changed during the ripening fermentation (from 4.26 ± 0.05 log cfu.g^−1^ and 4.38 ± 0.05 log cfu.g^‐−1^ to 4.61 ± 0.06 log cfu.g^‐−1^ and 4.57 ± 0.06 log cfu.g^−1^). Lactic acid bacteria increased from 6.32 ± 0.05 log cfu.g^−1^ in the initial phase to 7.57 ± 0.06 log cfu.g^−1^ and 7.64 ± 0.06 log cfu.g^−1^ in the end phase; and saccharomycetes increased from 6.47 ± 0.06 log cfu.g^−1^ in the initial phase to 8.57 ± 0.06 log cfu.g^−1^ and 8.68 ± 0.06 log cfu.g^−1^ in the end phase. In the control group, the number of lactic acid bacteria and saccharomycetes from 6.32 ± 0.05 log cfu.g^−1^ and 5.37 ± 0.05 log cfu.g^−1^ increased to 8.46 ± 0.08 log cfu.g^−1^ and 7.86 ± 0.07 log cfu.g^−1^, respectively, showing no significant difference from the starter culture group. However, APC was significantly higher for the control group than the starter culture group in the end fermentation phase, demonstrating substantial reproduction of other bacteria in the control group during fermentation. No monascus was detected in the control group, and no enterobacteria or pseudomonas was detected in the two groups. Increase in lactic acid bacteria and reproduction of saccharomycetes showed strong correlations (*p* < .05; *r* = 0.987) during the fermentation of Wanergao in the sample inoculated group and blank control group. In addition, lactic acid bacteria with highest dilution factor isolated from Wanergao in the starter culture group were identified as Lactobacillus plantarum by 16sRNA molecular biological technique, while lactic acid bacteria isolated in the control group were identified to include Lactobacillus plantarum, Pediococcus pentosaceus, and Lactobacillus brevis (as shown in Table 1). Meanwhile, saccharomycetes with highest dilution factor isolated from Wanergao in the starter culture group were identified as Saccharomyces cerevisiae by 26sRNA molecular biological technique, while saccharomycetes isolated in the control group included Saccharomycopsis fibuligera, in addition to Saccharomyces cerevisiae (as shown in Table 1). This result indicates that the red koji can well adapt to the fermentation environment of Wanergao. Not only monascus can be maintain stability, but also saccharomycetes can also grow fast in the acidic lactic acid bacteria‐forming environment utilizing lactic acid bacteria metabolites, while secreting metabolites required for growth of lactic acid bacteria without being affected by the antagonistic action of lactic acid bacteria and its metabolites (Zeng et al., 2017). In addition, Lactobacillus plantarum can make the most of nutrients in Wanergao, thereby holding advantage in the fermentation process. Through acid production and bacteriocins in fermentation process, the color, flavor, and texture of products are improved, product maturity are accelerated, and growth and reproduction of other spoilage bacteria and pathogenic bacteria are inhibited, thereby improving product quality and guaranteeing product safety.

**Table 1:** Sequence similarity of isolated strains with the reference sequences found in gene bank

Batches	Species	Source	% Identity
CN	*Lactobacillus plantarum*	KF673529	100
	*Pediococcus pentosaceus*	HG328247	99
	*Lactobacillus brevis*	FJ532360.1	99
	*Saccharomyces cerevisiae*	AJ746340	100
	*Saccharomyces cerevisiae*	CP011821.1	99
SM1	*Monascus purpureus*	HQ659499	99
	*Monascus ruber*	HQ857600	99
	*Lactobacillus plantarum*	KF673529	100
	*Saccharomyces cerevisiae*	AJ746340	100
SM2	*Monascus purpureus*	HQ659499	99
	*Monascus ruber*	HQ857600	99
	*Lactobacillus plantarum*	KF673529	100
	*Saccharomyces cerevisiae*	AJ746340	100

Note

Abbreviations: CN, no starter added; SM1, Like uncooked materials koji; SM2, Like liqueur koji.

### 3.2 Chemical analysis

**Figure 3:**
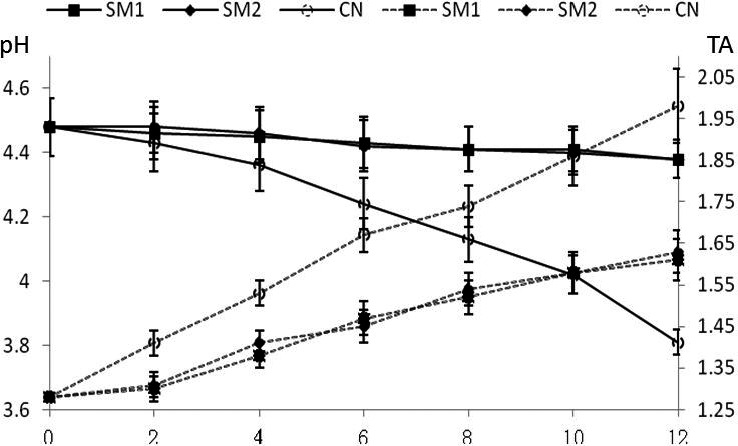
Changes in pH and TA values in Wangergao with/without starters duringfermentation. Opened round, CN: No starter added; Closed square, SM1 starter culture; Closed rhombus, SM2 starter culture

Changes in the pH and TA during fermentation of Wanergao are shown in Figure 3. Starter culture group exhibited lower pH decrease rate and lower TA increase rate than the control group during fermentation (*p* < .05). Within a fermentation time of 12 hr, pH dropped from 6.01 ± 0.04 in the initial phase to 4.44 ± 0.02 in the end phase in the starter culture group, while TA increased from 0.03% in the initial phase to 0.83% in the end phase. In the control group, pH dropped from 6.01 ± 0.04 in the initial phase to 3.87 ± 0.02 in the end phase, whereas TA increased from 0.03% in the initial phase to 1.23% in the end phase. This result shows that the control group Wanergao produced more acidic substances during fermentation process, thereby resulting in lower pH and increased TA. According to Wu (2009) report, rice partially produces lactic acid bacteria during soaking due to the pollution of environment, raw materials, and water resources; meanwhile, control group contains many acid‐forming bacteria such as acetic acid bacteria; with the progress of fermentation, reproduction of acid‐forming bacteria accompanies massive production of acidic substances, thereby resulting in lower pH and higher TA. In comparison, two red koji as the starter culture group mainly contains monascus, saccharomycetes, and a few other bacteria, so only minor amounts of acid‐forming bacteria were produced during fermentation. During fermentation, saccharomycetes and lactic acid bacteria are in an interdependent relationship. Saccharomycete reproduction needs to consume partial metabolites of lactic acid bacteria and thereby partially reduce the production of acidic substances (Zeng, Xia, Jiang, & Yang, 2013). Therefore, we speculate that more presence of acid‐forming bacteria causes lower pH and higher TA in the control group. In comparison, suitable pH and TA in the starter culture group impart soft acidity to products, which fits consumer demand. Similar conclusions have been widely reported in the research of sourdough fermentation. The presence of various types of lactic acid bacteria and saccharomycetes in the sourdough results in slightly decreased pH and slightly increased TA of products.

### 3.3 Characteristic analysis of rice slurry

Samples in the starter culture and control groups were subjected to characteristic analysis. Various characteristic indices are shown in Table 2.

**Table 2:** Protein, starch composition (%) and texture profile analysis for Wanergao

Parameters	Sample			
	BF SM1		SM2	CN
Protein (%)	5.75 ± 0.16^c^	3.68 ± 0.09^a^	3.77 ± 0.11^a^	4.37 ± 0. 11^b^
Total starch (%)	42.5 ± 1.27^a^	45.8 ± 1.75^bc^	44.7 ± 1.71^b^	48.6 ± 1.81^c^
Amylose (%)	10.6 ± 0.38^a^	14.8 ± 0.47^c^	15.2 ± 0.48^c^	13.4 ± 0.37^b^
Hardness (g)	4,138 ± 194^c^	2,154 ± 102^a^	2,127 ± 96^a^	2,396 ± 112^b^
Springiness (mm)	0.39 ± 0.01^a^	0.82 ± 0.02^c^	0.83 ± 0.02^c^	0.82 ± 0.03 ^b^
Cohesiveness	0.79 ± 0.01^c^	0.65 ± 0.02^a^	0.64 ± 0.02^a^	0.58 ± 0.01^b^
Gumminess (g)	151 ± 6.21^a^	316 ± 14.6^b^	307 ± 11.5^b^	311 ± 12.3^b^
Chewiness (g mm)	1,161 ± 38^a^	1,467 ± 48^c^	1,482 ± 45^c^	1,347 ± 31^b^
Adhesiveness	1968 ± 58^a^	1,141 ± 32^b^	1,139 ± 36^b^	1,143 ± 34^b^
Resilience	0.12 ± 0.01^a^	0.43 ± 0.01^c^	0.43 ± 0.01^c^	0.38 ± 0.01^b^

Note

Abbreviations: BF, before fermentation; CN, no starter added; SM1, Like uncooked materials koji; SM2, Like liqueur koji.

Values with unlike superscript letters in the same row are significantly different (*p* < .05) LSD test.

As can be seen from the table, protein content in rice slurry showed a more rapid decline in the starter culture group than in the control group during fermentation (*p* < .05). In the starter culture group, protein content dropped from 5.75 ± 0.16% in the initial phase to 3.68 ± 0.09% and 3.77 ± 0.11% in the end phase, while protein content in the control group was 4.37 ± 0.11% in the end phase of fermentation. Studies have shown that the red koji secretes protease degrading proteins during fermentation to form small molecular peptides and multiple amino acids, not only improving the protein digestibility, but also offering products good taste and flavor (Rahayu et al., 2017). Meanwhile, lactic acid produced during fermentation leads to a drop in pH, which will also result in protein dissolution.

During the fermentation process, total starch content decreased slightly in samples of all groups. Red koji‐inoculated samples exhibited slightly higher starch reduction than the control samples. The reason was presumably breakage and damage of partial rice grains caused during soaking process, which thereby increased the dissolution of soluble solids. Meanwhile, the presence of abundant amylase in the red koji degraded starchy substances in partial raw materials, resulting in slightly higher starch reduction in the samples of red koji‐inoculated group than the control group.

During the fermentation process, relative amylose content was slightly higher for the samples in the starter culture group than those in the control group. The reason was presumably the microcrystalline structure of amylopectin in rice was looser than the structure of amylose crystals, which was easily used by microorganisms (Liu, Cui, 2008), so starter culture group reproduced more monascus, saccharomycetes, and other microorganisms, which used amylopectins in a prioritized way to mildly degrade them, thereby lowering the amylopectin content. Meanwhile, protein and lipid degradation by monascus, saccharomycetes, and other microorganisms released the amylose bound to them, thus increasing amylose content in rice slurry. In addition, proteins and amylose in the rice presented mostly in a bound form, so we speculate that protein dissolution may also lead to increased amylose content.

### 3.4 Correlation analysis between characteristics of rice slurry and textural properties of Wanergao

In Table 2, amylose content was the one most influential to the quality of Wanergao among characteristic indices of rice slurry. Amylose content was significantly positively correlated with the hardness (*R* ^2^ = 0.849), cohesiveness, and resilience (*R* ^2^ = 0.947 and *R* ^2^ = 0.786, respectively), indicating that the higher the amylose content, the greater the hardness of Wanergao, and the stronger the cohesiveness and the better the resilience. Amylose content was significantly positively correlated with chewiness (*R* ^2^ = 0.794), which was consistent with the gel network model of rice starch. Amylose matrix ran throughout the gel network, which was the major constituent of gel. Thus, amylose content significantly influenced the quality of finished products. During the making of fermented rice products, rice proteins could not form a gluten network, and thus, the texture of Wanergao was maintained mainly by the gelatinized rice starch gel (Wu, 2009).

### 3.5 Analysis of fermentation performance of Wanergao

An important quality index for Wanergao was its aerogenic capacity during fermentation. In the experiment, fermenting power of red koji was estimated by measuring volume changes in three groups of Wanergao during fermentation. The results are shown in Table 3. Volume of Wanergao in the starter culture group increased from 100 ± 1.24 ml in the initial phase (0 hr) to 287.4 ± 4.53 ml and 294.4 ± 4.84 ml within a fermentation time of 12 hr, while the samples in the control group had a volume increase of 285 ± 4.49 mlafter fermenting for 12 hr. The results found no significant difference in volume increase between the starter culture and control groups (*p* > .05). This conclusion may be because saccharomycetes with strong ferment ability failed to hold advantage due to diversity of numerous saccharomycete species during Wanergao fermentation with red koji and backslopping, resulting in slow fermentation and unapparent manifestation of superior fermentation performance. Through numerous studies on sourdough and backslopping, researchers have concluded that aerogenic capacity reaches 260–290 only after fermenting with backslopping for 12–14 hr. This conclusion was very similar to ours (Shrivastava & Ananthanarayan, 2015).

**Table 3:** Batter volume and bulk density of Wanergao during fermentation

Fermentation period, hours	Rise in batter volume			Bulk density		
	SM1	SM2	CN	SM1	SM2	CN
0	100 ± 1.24^a^	100 ± 1.24^a^	100 ± 1.24^a^	0.71 ± 0. 02^a^	0.71 ± 0. 02^a^	0.71 ± 0. 02^a^
4	176 ± 3.26^ab^	181 ± 3.41^b^	168 ± 4.61^a^	0.94 ± 0.03^b^	0.97 ± 0.03^b^	0.91 ± 0.03^a^
8	231 ± 3.73^a^	235 ± 3.61^a^	233 ± 3.43^a^	1.16 ± 0.05^b^	1.17 ± 0.06^b^	1.13 ± 0.04^a^
12	287.4 ± 4.53^a^	294.4 ± 4.84^a^	285 ± 4.49^a^	1.47 ± 0.07	1.51 ± 0.08	1.43 ± 0.07

Note

Abbreviations: CN, no starter added; CN, no starter added; SM1, Like uncooked materials koji; SM2, Like liqueur koji.

Values with unlike superscript letters in the same row are significantly different (*p* < .05) LSD test.

Densities of Wanergao samples in the three groups are shown in Table 3. Fermentation end products of Wanergao in the two groups revealed similar density values (*p* > .05). Samples in the three groups all needed 12 hr to achieve good fermentation effects, forming substantially equal volume and density. Density was significantly correlated with the hardness and volume of fermented rice products. When the product hardness was high and volume growth was small, density was also high correspondingly and vice versa. Our experiment showed that Wanergao in both the starter culture and control groups can form substantial gas to increase product volume during fermentation, thus giving Wanergao soft texture and small density. Wu(2009) also stated in the research on specific volume of rice‐steamed sponge cakes that smaller density can offer products better elasticity, higher resilience, and suitable chewiness.

### 3.6 Analysis of free amino acids

Free amino acid compositions of raw rice, starter culture‐inoculated Wanergao, and control Wanergao are shown in Table 4.

**Table 4:** Changes in free amino acids of Wanergao during fermentation

Free amino acid (mg/g)	Sample			
	BF	SM1	SM2	CN
Asp	3.01 ± 0.11^a^	5.37 ± 0.24^b^	5.57 ± 0.23^b^	4.37 ± 0.21^b^
Thr	1.67 ± 0.09^a^	2.27 ± 0.05^c^	2.22 ± 0.08^c^	2.19 ± 0.05^b^
Ser	2.06 ± 0.06^a^	2.54 ± 0.08^b^	2.61 ± 0.07^b^	2.52 ± 0.06^b^
Glu	6.61 ± 0.31^a^	7.76 ± 0.28^c^	7.81 ± 0.26^b^	7.92 ± 0.27^b^
Gly	1.82 ± 0.07^a^	2.36 ± 0.07^c^	2.39 ± 0.08^c^	2.02 ± 0.06^b^
Ala	2.12 ± 0.07^a^	3.42 ± 0.17^c^	3.21 ± 0.13^b c^	3.32 ± 0.11^b^
Cys	1.68 ± 0.07^a^	2.61 ± 0.11^c^	2.54 ± 0.11^c^	2.68 ± 0.12^b^
Val	2.86 ± 0.11^a^	4.86 ± 0.16^c^	4.68 ± 0.16^c^	3.68 ± 0.12^b^
Met	0.96 ± 0.02^a^	1.37 ± 0.03^b^	1.29 ± 0.03^b^	1.33 ± 0.03^ab^
Ile	1.92 ± 0.07^a^	2.42 ± 0.11^c^	2.28 ± 0.11^bc^	2.53 ± 0.12^b^
Leu	3.52 ± 0.18^a^	4.89 ± 0.23^b^	5.03 ± 0.24^b^	4.53 ± 0.22^b^
Tyr	2.34 ± 0.11^bc^	2.54 ± 0.13^b^	2.61 ± 0.13^b^	2.11 ± 0.12^a^
Phe	3.14 ± 0.14	3.46 ± 0.16	3.38 ± 0.12	3.44 ± 0.15
Lys	2.01 ± 0.11	2.24 ± 0.11	2.26 ± 0.12	2.16 ± 0.11
His	1.47 ± 0.04^a^	2.16 ± 0.06^b^	2.21 ± 0.07^b^	2.26 ± 0.08^b^
Arg	4.01 ± 0.12^a^	5.26 ± 0.18^c^	5.39 ± 0.17^bc^	5.24 ± 0.17^b^
Pro	1.28 ± 0.06^a^	1.57 ± 0.07^c^	1.62 ± 0.07^c^	1.69 ± 0.07^b^
TAA	41.6 ± 1.32^a^	57.1 ± 2.12^c^	57.2 ± 2.17^bc^	54.1 ± 2.37^b^
EAA	15.2 ± 0.63^a^	21.5 ± 1.13^b^	21.1 ± 0.91^b^	19.8 ± 0.92^b^
DAA	19.1 ± 0.71^a^	24.9 ± 1.48^bc^	27.1 ± 1.82^c^	23.1 ± 1.08^b^
NEAA	24.9 ± 1.15^a^	33.4 ± 1.62^c^	33.9 ± 1.52^bc^	32.1 ± 1.48^b^
EAA/ TAA (%)	36.5 ± 1.23^b^	37.6 ± 1.28^a^	36.8 ± 1.39^a^	36.6 ± 1.24^a^
DAA/ TAA (%)	45.9 ± 1.51^b^	43.6 ± 1.37^a^	47.3 ± 1.42^a^	42.7 ± 1.26^a^
EAA/ DAA	0.79 ± 0.03	0.86 ± 0.03	0.78 ± 0.02	0.86 ± 0.03

Note

Abbreviations: BF, before fermentation; CN, no starter added; CN, no starter added; DAA, delicious amino acid; EAA, essential amino acid; NEAA, nonessential amino acid; SM1, Like uncooked materials koji; SM2, Like liqueur koji; TAA, total amino acids.

Values with unlike superscript letters in the same row are significantly different (*p* < .05) LSD test.

Table 4 shows total amino acids (TAA), essential amino acids (EAA), delicious amino acids (DAA), and nonessential amino acids (NEAA) in samples. Contents of free amino acids contained in starter culture‐inoculated Wanergao were all higher than those in the control Wanergao and raw rice. Contents of aspartic acid, glycine, valine, and flavoring leucine were all several times higher than the control Wanergao and raw rice.

This was because enzymes secreted by red koji during fermentation of Wanergao in the starter culture group can partially degrade macromolecular proteins to form small molecular peptides, free amino acids, and other flavor substances (Rojas & Stein, 2013). Meanwhile, during fermentation of Wanergao, free amino acids and peptides from self‐production of saccharomycetes can also supplement nutritional deficiencies of Wanergao, thereby giving Wanergao authentic taste.

In this study, after fermenting Wanergao with red koji and backslopping for 12 hr, EAA and DAA contents significantly elevated compared with the raw rice group (*p* < .05), contributing greatly to the flavor and nutrition formation of Wanergao. For sulfur‐containing amino acids often lack in dairy products, such as methionine and cysteine, their contents increased markedly in the fermented Wanergao. Methionine can increase from 0.96 ± 0.02 mg/100 g before fermentation to 1.29 ± 0.03–1.37 ± 0.03 mg/100 g, whereas cysteine can increase from 1.68 ± 0.07 mg/100 g before fermentation to 2.54 ± 0.11–2.68 ± 0.12 mg/100 g. Inclusion of such foods in daily diet can make up for lack of amino acids such as methionine and cysteine.

### 3.7 Citrinin analysis

Citrinin is a secondary metabolite produced by fungi. As a fungal toxin, it can cause a series of symptoms such as renal tubular ectasia, epithelial cell degeneration and necrosis, kidney swelling, and copious urine in experimental animals (Rojas & Stein, 2013). French professor (Suharna, 2015) stated that the majority of monascus strains can produce citrinin, so the safety of red yeast rice has gained wide public attention.

In this study, no citrinin was detected in three types of Wanergao samples during fermentation. Studies have found that citrinin production was closely associated with monascus species and production processes (Wang, Chen, Wang, Li, & Wang, 2011). Citrinin tends to increase gradually with prolonged fermentation time. According to Castellá's report, citrinin was often produced gradually 2–3 d after fermentation in fermented foods (Castellá, Cabañes, Bragulat, & Martínez, 2008). Starter culture group Wanergao was ripened by cooking, and further, fermentation of red yeast rice was terminated only 12 hr after fermentation. Thus, we speculate that during the fermentation of Wanergao by red yeast rice, fermentation ended before monascus produced any citrinin.

### 3.8 Color value analysis

Red koji as a natural colorant and starter culture has advantages unparalleled by many other natural colorants such as pH stability, high capacity strong fermentation, and heat resistance. The most important secondary metabolites of Monascus spp., mainly included yellow pigments (ankaflavin and monascin), orange pigments (monascorubrin and rubropunctatin), and red pigments (monascorubramine and rubropunctamine) (Liu et al., 2019), which might be contribute attractive color in some food.

Changes in color value (CV) of starter culture group Wanergao during fermentation are not shown. In the two starter culture groups, CV did not change much during the ripening and fermentation phase. No CV was detected in the control group. The reasons might be explained as monascus grows white mycelium about 48 hr after culture, and the colony color does not appear to be red or orange‐red until 5 days after culture, while the fermentation time was only 12 hr in the two starter culture group, which secrete minor amounts of secondary metabolites, that is, rare production of colorants. However, the fermentation time was up to 10–15 days in koji rice wine, mycelia did secrete large amounts of coloring substances along with monascus reached a certain number. Therefore, the color of the Wanergao owes more to the effect of the koji rice wine than to the color produced by the growth of monascus.

### 3.9 Texture profile analysis (TPA)

TPA of finished Wanergao samples with a texture analyzer is shown in Table 2. TPA indices of Wanergao samples consisted of: hardness, cohesiveness, gumminess, chewiness, resilience, springiness, and adhesiveness. As shown in Table 2, Wanergao samples in the starter culture group exhibited significant differences in chewiness and hardness from control samples (*p* < .05), while presenting no significant difference in springiness or adhesiveness (*p* > .05). The differences in hardness and chewiness between the starter culture and control group samples were presumably because the massive enzymatic substances contained in red koji degraded amylopectins, proteins, and other non‐sugar substances in rice slurry, resulting in increased relative amylose content, and thereby improved the hardness and chewiness of products. No significant difference in springiness between the starter culture and control group samples indicated similar CO_2_ production capacities by saccharomycete growth and reproduction during fermentation in the two groups. Cohesiveness was significantly higher for the starter culture group than the control group (*p* < .05). Cohesiveness represented the ability to resist shear, denteness. Starter culture group samples showed higher cohesiveness, suggesting high resilience and chewiness. Resilience was significantly higher for the starter culture group than the control group, indicating that Wanergao in the starter culture group were more aldente and chewy. No significant difference was observed in adhesiveness between the three groups (*p* > .05); low adhesiveness meant that Wanergao did not stick to the teeth.

### 3.10 Sensory analysis

**Figure 4:**
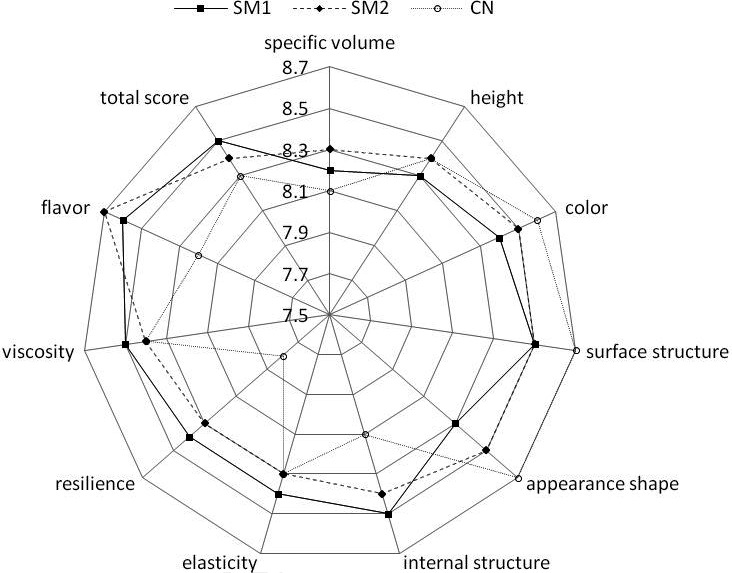
Sensory characteristics Wangergao with/without starters. Opened round, CN: No starter added; Closed square, SM1 starter culture; Closed rhombus, SM2 starter culture

Sensory evaluation results of the three types of Wanergao samples are shown in Figure 4. As can be seen from the figure, Wanergao in the two starter culture group showed significantly different surface color from the control group (*p* < .05). No significant difference in scores of four indices, that is, surface structure, appearance shape, internal structure, or elasticity, was observed between the two groups (*p* > .05). Flavor score of Wanergao in the starter culture group was 8.6 and 8.7, which was higher than 8.2 of control samples. Red koji produced large amounts of enzymes such as glucoamylase, amylase, and protease in the fermentation process, thereby imparting unique aroma to the products (Becker, French, Morris, Silvent, & Gordon, 2013). Comparison of specific volume, height, and total score between the starter culture‐ and backslopping‐inoculated Wanergao (see Table 3) demonstrated no significant difference in specific volume (*p* > .05). Both the red koji and backslopping‐inoculated Wanergao exhibited long fermentation time, inadequate yeast growth capacity during fermentation and insufficient fermentation activity. In addition, all groups of samples had complex microbial composition. During the fermentation process, other non‐yeast microorganisms may have some impact on yeast fermentation, so differences in specific volume and fermentation height were not significant between the three groups. As for the total score, the three groups showed almost the same scores, which was attributable to great difference in each index and large deviation in sensory evaluation. Total sensory evaluation score demonstrated that the red koji and backslopping‐inoculated Wanergao had respective advantages and disadvantages in terms of quality, which was similar to the results of the sensory evaluation.

Elasticity, viscosity, and resilience in the sensory evaluation were somewhat similar to the springiness, adhesiveness, and cohesiveness in the texture analyzer, which also had some consistency with the differential evaluation of relevant indices of three types of Wanergao. High‐quality Wanergao should have good chewiness, small viscosity, good elasticity, and nice aroma. In summary, this study demonstrated that the red koji‐fermented Wanergao had good chewiness, high resilience, high hardness, nice aroma, and good color. However, they exhibited no significant difference in viscosity or elasticity from the control samples.

## 4 CONCLUSION

Wanergao is a traditional fermented rice product. There was no significant difference in batter volume within 12 hr of fermentation between the starter group and the control group (*p* > .05). Lactic acid bacteria (mainly Leuconostoc lactis) and saccharomycetes (mainly Saccharomyces cerevisiae) grow substantially in the two red koji‐inoculated Wanergao during fermentation, resulting in gradually declining pH and gradually increasing TA, and thereby form unique soft acidity of Wanergao. Compared to the Wanergao made using traditional backslopping, the red koji‐inoculated Wanergao also contain more free amino acids and amylases, giving Wanergao rich nutrition, flavor, and unique gel properties. In addition, red koji‐inoculated Wanergao also present high textural parameters such as aroma, resilience, chewiness, cohesiveness, and hardness.

## AUTHOR CONTRIBUTIONS

Xuefeng Zeng and Zhongyue Tang designed and conducted the research and wrote the main manuscript text. Jin Fan directed the research, reviewed, and revised the manuscript. Laping He, Li Deng, and Chun Ye modified articles.
